# Complete coding sequence characterization and comparative analysis of the putative novel human rhinovirus (HRV) species C and B

**DOI:** 10.1186/1743-422X-8-5

**Published:** 2011-01-07

**Authors:** Piyada Linsuwanon, Sunchai Payungporn, Kamol Suwannakarn, Thaweesak Chieochansin, Apiradee Theamboonlers, Yong Poovorawan

**Affiliations:** 1 Center of Excellence in Clinical Virology, Department of Pediatrics, Faculty of Medicine, Chulalongkorn University and Hospital, Bangkok, Thailand; 2 Department of Biochemistry, Faculty of Medicine, Chulalongkorn University and Hospital, Bangkok, Thailand

## Abstract

**Background:**

Human Rhinoviruses (HRVs) are well recognized viral pathogens associated with acute respiratory tract illnesses (RTIs) abundant worldwide. Although recent studies have phylogenetically identified the new HRV species (HRV-C), data on molecular epidemiology, genetic diversity, and clinical manifestation have been limited.

**Result:**

To gain new insight into HRV genetic diversity, we determined the complete coding sequences of putative new members of HRV species C (HRV-CU072 with 1% prevalence) and HRV-B (HRV-CU211) identified from clinical specimens collected from pediatric patients diagnosed with a symptom of acute lower RTI. Complete coding sequence and phylogenetic analysis revealed that the HRV-CU072 strain shared a recent common ancestor with most closely related Chinese strain (N4). Comparative analysis at the protein level showed that HRV-CU072 might accumulate substitutional mutations in structural proteins, as well as nonstructural proteins 3C and 3 D. Comparative analysis of all available HRVs and HEVs indicated that HRV-C contains a relatively high G+C content and is more closely related to HEV-D. This might be correlated to their replication and capability to adapt to the high temperature environment of the human lower respiratory tract. We herein report an infrequently occurring intra-species recombination event in HRV-B species (HRV-CU211) with a crossing over having taken place at the boundary of VP2 and VP3 genes. Moreover, we observed phylogenetic compatibility in all HRV species and suggest that dynamic mechanisms for HRV evolution seem to be related to recombination events. These findings indicated that the elementary units shaping the genetic diversity of HRV-C could be found in the nonstructural 2A and 3D genes.

**Conclusion:**

This study provides information for understanding HRV genetic diversity and insight into the role of selection pressure and recombination mechanisms influencing HRV evolution.

## Introduction

Human rhinoviruses (HRVs) are one of the most highly prevalent ethological agents of acute respiratory tract illness (RTI) and, among other factors, contribute to children's hospitalization and morbidity. The clinical manifestations associated with HRV infection are predominantly asymptomatic or self-limited upper RTIs with a short incubation period of 1 to 3 days, similar to a common cold or influenza-like illnesses. Several studies have recently reported that HRV infection in children can also be associated with numerous clinical illnesses, contributing to acute exacerbations and inflammatory respiratory diseases. Among these are acute community-acquired sinusitis [1,2], community-acquired pneumonia [3,4], chronic obstructive pulmonary disease exacerbation [5-7], bronchiolitis [8,9], wheezing [10-12], and asthma exacerbation [13-15]. However, the association of HRV infection with exacerbation and the pathogenic mechanisms by which HRVs directly influence more severe RTIs are not well established.

HRVs are small, non-enveloped viruses of 30 nm diameter classified in the genus *Enterovirus *of the diverse family *Picornaviridae*. The highly structured icosahedral capsid contains a single-stranded RNA genome of positive polarity approximately 7,200 base pairs (bp) in length. Similar to their close relative, human enterovirus (HEV), the coding sequences comprise 4 structural genes, VP1-VP4, and 7 non-structural genes. These non-structural genes are translated in the cytoplasm of the infected cell to produce a single polyprotein precursor of approximately 2,200 amino acid residues, and are immediately cleaved upon synthesis of virus encoded protease. HRVs can replicate in airway epithelial cells of both the upper and lower respiratory tract. Acid intolerance prevents HRV replication in the gastrointestinal tract and thus differentiates them from other enteroviruses.

HRVs display genetic and antigenic variability. Hence, based on immunology they have been historically classified into 99 reference serotypes correlated with serological neutralization activity. HRVs can also be categorized by several parameters, including receptor specificity (ICAM-1 and LDL-R) and antiviral drug susceptibility. Recent molecular techniques have applied bioinformatics methods to analyze their evolutionary relationships based on sequence compatibility of 5'UTR or partial capsid genes. Capsid genes commonly focused on include the VP1 region, which has been reported to be an essential part of the viral neutralization antigenic determinant to evade the host's immune response and is utilized as a binding site of synthetic antiviral compounds [16-19], or the VP4 or VP4/2 genes. Based on these techniques, all reference serotypes have been divided into 3 species, comprising 2 previously defined species, HRV-A (n = 74), and HRV-B (n = 25) [18], and the new species HRV-C (33 types proposed based on VP1 gene) [20-23].

Recently, several epidemiological studies based on PCR amplification have reported that HRV-C was more predominantly found in pediatric patients hospitalized with acute lower RTI [21,24,25] as compared to other HRVs. HRV-C has thus been proposed as an etiological agent associated with recurrent wheezing [11,26] and asthma exacerbation [13-15,26] which might not be susceptible to appropriate antibiotic treatment. However, the inability to grow HRV-C in tissue culture has limited the understanding of their pathogenicity and the mechanisms of host immune response to HRV-C infection.

As part of the retrospective epidemiological exploration of common respiratory viruses in Thailand during February 2006-2007, a total of 87 nasopharyngeal (NP) suction specimens from 289 samples were found infected with HRV. Phylogenetic classification established the high diversity of HRV and predominance of species C in Thailand [24]. To further explore the genetic characteristics, clinical impact, and evolutionary divergence of HRV species, we have extended our previous research by characterizing the full-length coding sequence of the 6 representative HRV strains circulating in Thailand and report the discovery of putative new HRV-C and HRV-B strains. Moreover, we have comparatively analyzed all HRV prototypes in order to elucidate the occurrence of recombination in each of the HRV species.

## Methods

### HRV positive specimens and viral nucleic acid preparation

The NP suction specimens were collected from pediatric patients hospitalized at King Chulalongkorn Memorial Hospital, Thailand between February 2006 and 2007. Admission criteria of the study population were based on clinical presentations combined with other laboratory results as described in previous reports. RNA was extracted from stored samples and then cDNA was synthesized as described elsewhere [24].

### PCR amplification and nucleotide sequencing

Primer sets for HRV entire coding sequence amplifications were designed based on each species' specific nucleotide sequence available at the GenBank database (primer sequences upon request). The sequences of the genome termini were arrived at by a specific PCR technique developed from a modified 3'RACE method [27]. All purified PCR products were bidirectionally sequenced with the 2 primers used in the second round of semi-nested PCR provided by First BASE Laboratories Sdn Bhd (Selangor Darul Ehsan, Malaysia).

### Complete coding sequence analyses

Sequences were prepared and aligned using Clustal W implemented in the BioEdit program version 7.0.4.1 http://www.mbio.ncsu.edu/BioEdit/bioedit.html. A Pairwise Sequence similarity plot was calculated and depicted using SimPlot software [28] with Jukes-Cantor parameter, window size of 400 bp and a step size of 20 bp. To examine the picornaviral protease cleavage sites (2A^pro^, 3C^pro^, autocatalytic sites), sequences were sought using the Net-PicoRNA 1.0 server [29]. Consensus cis-acting replication element (*cre*) sequences of the selected alignment regions were evaluated using the RNAalifold [30] and MFold server [31].

### Phylogenetic analyses

To determine the phylogenetic relationship between HRV complete coding sequences and their polyprotein, the phylogenetic tree was constructed by using the neighbor-joining method with Kimura's two-parameter substitution model. Data was bootstrap re-sampled 1,000 times for nodal confidence value determination implemented in the MEGA version 4.0 program package [32].

### Phylogenetic compatibility matrix

Phylogenetic compatibility matrix (PCM) analysis is a computational method used to investigate the phylogenetic relationship of the sequences to be analyzed. The PCM plot of nucleotide sequence alignment in intra- and inter-HRV species was constructed by using the program TreeOrderScan in the Simmonic 2007 version 1.6 [33]. All published HRV reference nucleotide sequences of each species including 75 HRV-A, 25 HRV-B, 9 HRV-C, and our 6 identified strains were aligned and computed separately between and within species using the programs SEQBOOT, DNADIST, NEIGHBOR-JOINING and PHYLIP with the following program setting: 250 bp fragment length, 100-bp increments, 100 fold resampling with 70% bootstrap threshold value that subsequently generated 65 aligned fragments of HRV-A and HRV-B while HRV-C was generated from 64 overlapping fragments.

### Recombination analysis

Potential recombination events within the coding regions were assessed using phylogenetic analysis based on the various viral genome parts with high recombination rate. To confirm an accurate recombination event, the complete coding sequences were analyzed in comparison with all known reference sequences by using the Recombination Detection Program 3Beta41 [34]. Manual Bootscanning was performed by using Jukes-Cantor algorithm and neighbor-joining method [27,35,36] with a parameter setting of 200 bp window size, 10 bp step size and 1,000 bootstrap replicates.

### G+C content analysis

To analyze the G+C content of the full-length coding sequences of each HRV species, a total of 20 HRV-A, 25 HRV-B, and all HRV-C coding sequences available at the GenBank database were selected. Three representatives of each HEV species as well as 3 distinct Polioviruses were chosen from the database under the following accession numbers: HEV-A (DQ452074, AY421760, and AY421769), HEV-B (AF241359, AF081485, and AF029859), HEV-C (NC_001428, AF499640, and AF499635), HEV-D (NC_001430, EF107098, and DQ201177), and Polioviruses (V01150, X00595, and X00925). The GC percent composition was directly compared within the viral reading frame and plotted with standard deviation using online software including CpG ratio/GC content http://mwsross.bms.ed.ac.uk/public/cgi-bin/cpg.pl and GC content/GC skew diagrams http://nbc11.biologie.unikl.de/framed/left/menu/auto/right/GC/ with a parameter setting of 500 bp sliding window and 10 bp increment size between successive windows.

## Results

### Complete coding sequence analysis

**Figure 1:**
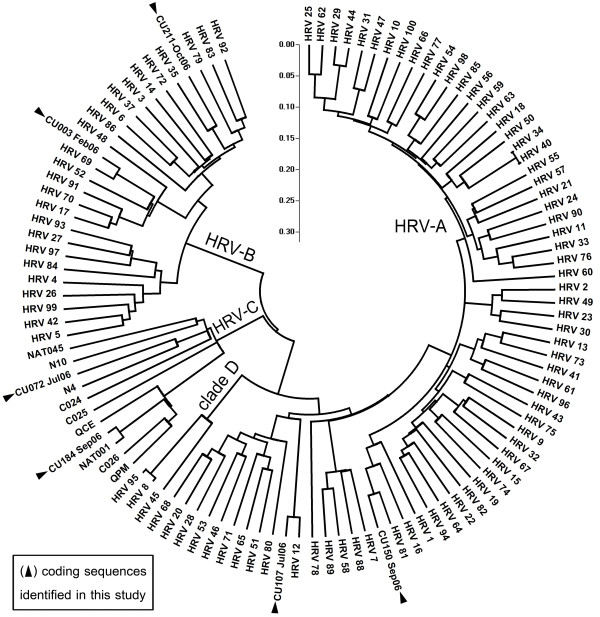
**Phylogenetic analysis illustrating genetic relationships between HRV species based on sequence alignment of 6 complete coding sequences amplified from our study (black triangle) compared with all known HRV prototypes**. The neighbor-joining phylogenetic tree was constructed using Kimura's two-parameter with 1,000 bootstrap replicates using the MEGA4 program. Evolutionary distance was represented by the scale bar in the unit of nucleotide substitutions per site. The selected HRV strain name in this study refers to number of specimen and patient's admission month and year.

**Figure 2:**
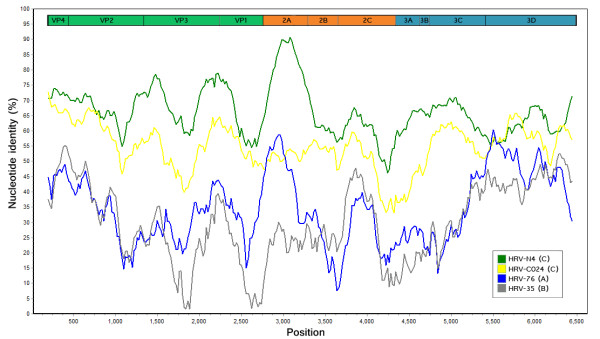
**Complete coding sequence similarity plot illustrating pairwise sequence identity between HRV-CU072 compared with the most closely related Chinese strain (N4; green line) and other HRV members (HRV-C024; yellow line, HRV-76; blue line, HRV-35; gray line)**. Constructed using SimPlot v3.2 with Jukes-Cantor parameter, window size of 400 bp and a step size of 20 bp, and 1,000 bootstrap replicates.

**Figure 3:**
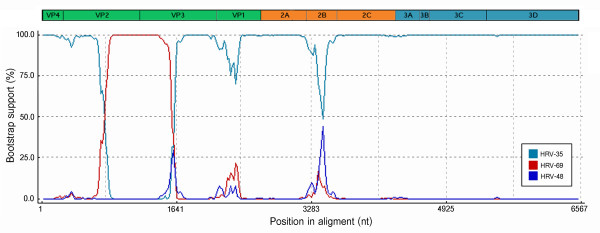
**A Bootscanning plot of recombination between the daughter strain HRV-CU211 and major (HRV-35) or minor (HRV-69) parental strains**. Recombination breakpoint was predicted to occur at the ORF's nucleotide positions 766-1,590 covering partial VP2 and VP3 capsid encoding genes. Bootstrapping support value was computed using the RDP3 program with a window size of 200 bp, step size of 10 bp, and 1,000 bootstrap replicates.

The entire coding sequences of the 6 additional HRV strains elucidated in this study have been submitted to the GenBank database and assigned accession numbers HQ123440-HQ123445. Nucleotide and deduced amino acid sequence analysis revealed considerably different phylogenetic clustering features of the strains HRV-CU072 (HQ123440) and HRV-CU211 (HQ123444) as showed in Figure 1. The strain HRV-CU072 displayed relatively low pairwise sequence identity compared with other HRV-Cs (66%) (Figure 2). Furthermore, scanning bootstrap analysis supported our finding that the strain HRV-CU211 is a putative new HRV strain derived from intra-species recombination of HRV-B (Figure 3).

The HRV-CU072 coding sequence spanned 6,450 nt region rich in A and U bases and encoded a 2,149 aa polyprotein. Similar to other HRV-C members, HRV-CU072 had a relatively small polyprotein gene due to a deletion in the major part of the antigen neutralization site covering the BC, DE, and HI loops of the VP1 protein and shared 50% and 45% amino acid sequence identity with HRV-A and HRV-B, respectively. Direct investigation of the VP1 gene revealed that HRV-CU072 shared only 64% sequence identity with the other HRV-Cs.

### HRV-CU072 coding sequence analysis

To investigate the molecular characteristics of the putative new HRV-C strain, we performed comparative analysis of the HRV-CU072 complete coding sequence with all available HRV references and the representative members of different HEV species. An alignment of deduced amino acid sequences was generated allowing for the 10 hypothetical cleavage sites of the HRV-CU072 polyprotein (Table 1). In addition, half of all cleavage sites of the HRV-CU072 strain's conserved amino acid residues were commonly found in HRV members while some cleavage site features, such as an identical M/S pair in the autocatalytic cleavage site between the structural proteins VP4 and VP2, were also found in HRV-CU072 and other HRV-Cs. The unique amino acid sequences of HRV-CU072's protease cleavage site were observed at the VP3/VP1 site as N/D residues while other HRV-C members utilized an alternative cleavage Q/N pair similar to HRV-As. However, amino acid polarity remained unchanged.

**Table 1:** Amino acid residues within viral-encoded protease cleavage sites of the HRV-CU072 polyprotein compared with putative sites of other HRV species.

protein junction	CU072	HRV-C	HRV-A	HRV-B
VP4/VP2	M/S	M/S	Q/S	N/S
VP2/VP3	Q/G	Q/G	Q, E/G	Q/G
VP3/VP1	N/D	Q/N	Q/N	E/G
VP1/2A	V/G	A, L/G	A, F, V, Y/G	Y/G
2A/2B	Q/G	Q/G	Q/G	Q/G
2B/2C	Q/S	Q/G, S	E, Q/S	Q/A, S
2C/3A	Q/G	Q/G	Q/G	Q/G
3A/3B	Q/G	Q/G	Q/G	Q/G
3B/3C	Q/G	Q/G	Q/G	Q/G
3C/3D	Q/G	Q/G	Q/G	Q/G

An estimated sequence variation was calculated using pair-wise nucleotide and deduced amino acid sequence alignment and indicated as a percentage of each individual viral protein.

Comparison of HRV-CU072's individual protein products with other HRV-C members showed that the VP4 protein was a highly conserved protein among other HRV and HEV species. Similar with other HRV-C members, HRV-CU072 displayed a cis-acting replication element (*cre*: **R_1_**NNN**AAR_2_**NNNNNN**R_3_**) as **G**CUU**AAA**CAAAUU**A **located in the VP2 protein different from HRV-As and HRV-Bs where the *cre *structure is located in the 2A and 2C region, respectively. The G(P/A)Y(S/T)GxP motif within the 3B protein (VPg) crucial for phosphodiester linkage formation between the VPg protein and 5'end of viral RNA was identified in the HRV-CU072 sequence. Furthermore, at position 4 of this motif, almost all HRV-C members displayed the unique Thr residue while only strains HRV-CU072, C025 (EF582386), N4 (GQ223227), and N10 (GQ223228) shared the conserved Ser residue in common with HRV-A and HRV-B.

To determine cell-specific receptor usage (major receptor = ICAM-1 and minor receptor = LDL-R), conserved motif and functional domain of the HRV-CU072 strain, the deduced amino acid sequences of protein VP1 and carboxy-terminal VP3 were aligned. In total, 5 of 9 and 4 of 7 conserved residues corresponding to the ICAM-1 footprint of the HRV-A and HRV-B major group members, respectively, were found in the HRV-CU072 strain. The fully conserved residue Gly1148 shared between the HRV-A/major and HRV-A/minor group was also identified in the HRV-CU072 strain. The key residue Lys224 within the TEK motif located in the VP1 protein essential for rhinovirion and LDL-R protein interaction [37] was not found in HRV-CU072. An 8-10 amino acid insertion found in HRV-CU072's VP1 sequence represented some characteristics unique from other HRV members, such as a hydrophilic amino acid insertion in the GH loop. Furthermore, the HRV-CU072 strain might be resistant to pleconaril due to amino acid substitutions in the 2 positions (152 and 191) crucial for identifying naturally resistant serotypes [38] located in the drug binding pocket identified as Y52F and V191T.

### Comparative analysis of the HRV-CU072 strain with most closely related strains

To elucidate the genetic relationship between the HRV-CU072 strain and other HRV-Cs, an estimated amount of synonymous (S) and nonsynonymous (NS) variation at the protein level was investigated (Table 2). In this analysis, nonsynonymous changes were defined as 2 types of variation: nonconservative (NC-NS) and conservative nonsynonymous (C-NS) variation and were based on the presence or absence of changes in amino acid polarity, respectively. Sequence comparison of each individual protein precursor between HRV-CU072 and its closest relative (China's strain N4: GQ223227) indicated that the VP4 and 3A proteins showed the highest overall sequence identity score (87%) whereas the VP2 protein represented the least conserved protein among them. The VP2 region was found to have the largest numbers of both amino acid sequence variation (31%) and NS variation (58%) while the 3A region exhibited the lowest amino acid sequence variation (12%). Even though the 2A protein had less NS variation than the VP2 (41%), this protein displayed the highest percent NC-NS variation (48%). While the lowest NS score was found in the 2C region (19%), this region had undergone profound NC-NS evolutionary change (44%) compared to other regions. Overall, the structural proteins of the HRV-CU072 strain, especially in the proteins VP1-3, showed a high average of NS variability compared to the N4 strain.

**Table 2:** Evolutionary relationship along ORF of HRV-CU072 compared with the most closely matched N4 strain.

	Structural proteins				Nonstructural proteins						
	
Viral protein	VP4	VP2	VP3	VP1	2A	2B	2C	3A	3B	3C	3D
Variation (nt)	55	209	176	213	81	63	316	59	20	139	397
Nucleotide variation (nt%)	27	26	25	26	19	21	32	26	30	25	29
Amino acid variation (aa%)	14	31	25	23	16	19	26	12	23	16	24

NS variation (aa)	9	80	56	63	23	19	34	9	5	29	70
NS variation (%)	19	58	42	44	41	43	19	20	36	25	30
NC-NS variation (%NC)	33	40	38	29	48	21	44	33	40	14	30

NS = nonsynonymous, NC = nonconservative amino acid

### Phylogenetic relationship

**Figure 4:**
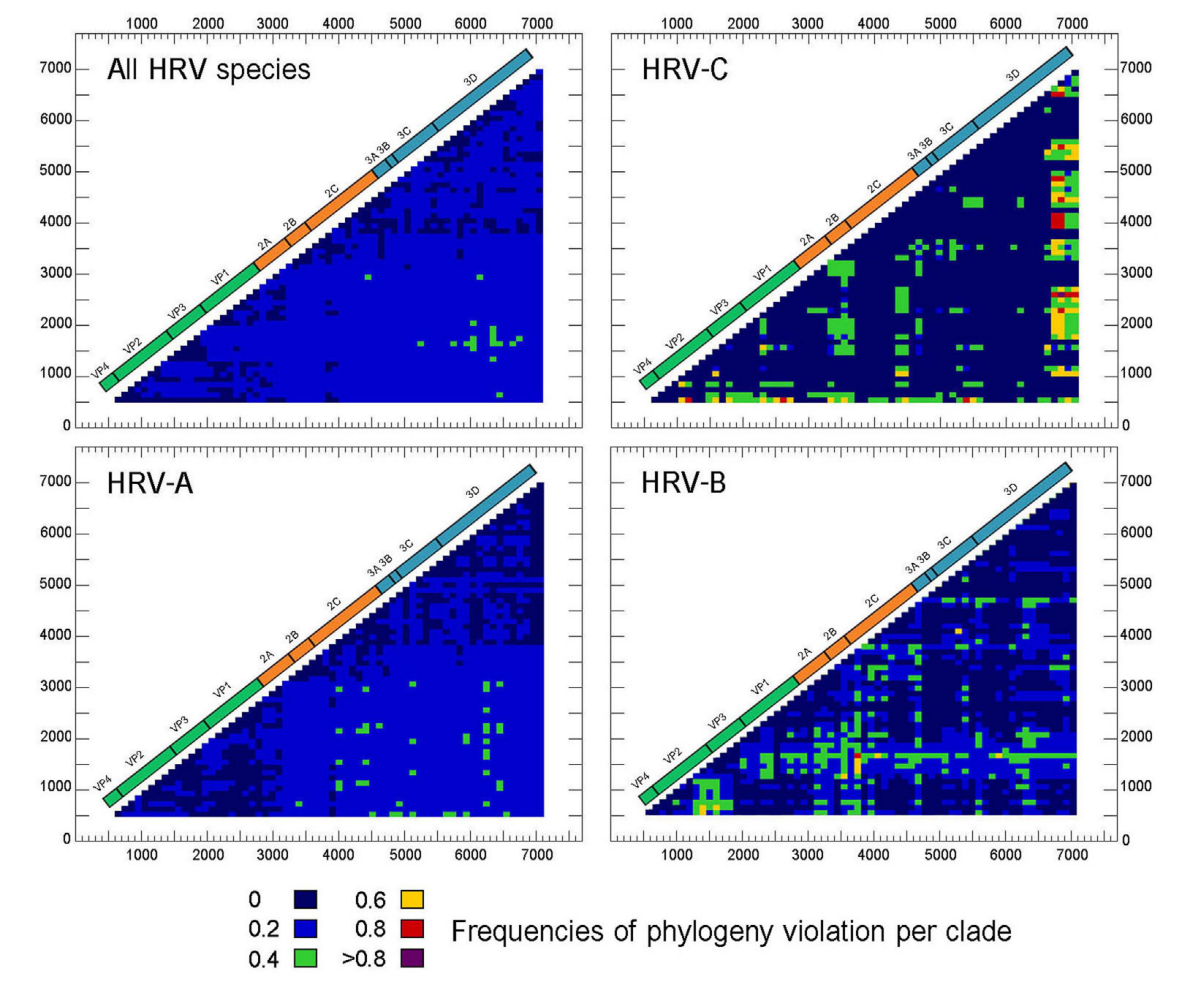
**Phylogenetic compatibility matrices of HRV species A, B, and C. Multiple sequence alignments of all known HRV prototypes including 6 identified sequences derived from our study were individually performed using TreeOrderScan program (Simmonds and Smith, 1999)**. The numbers of phylogeny violation are color coded corresponding to an incompatibility frequency score of pairwise fragment comparison.

To observe changes in phylogenetic relationships, the PCM plot of nucleotide sequence alignment was performed using the program TreeOrderScan. The PCM results of each HRV species are summarized in Figure 4. HRV-As showed the lowest degree of phylogenetic incompatibility throughout the coding region, which correlated to a high level of sequence identity. The frequency of recombination in HRV-B and HRV-C was shown to be higher than HRV-A. HRV-C's phylogenetic relationship among species members had altered in the 2A and at the 3' terminal of 3D coding regions while the remaining genome regions remained conserved.

### Recombination detection in HRVs

**Figure 5:**
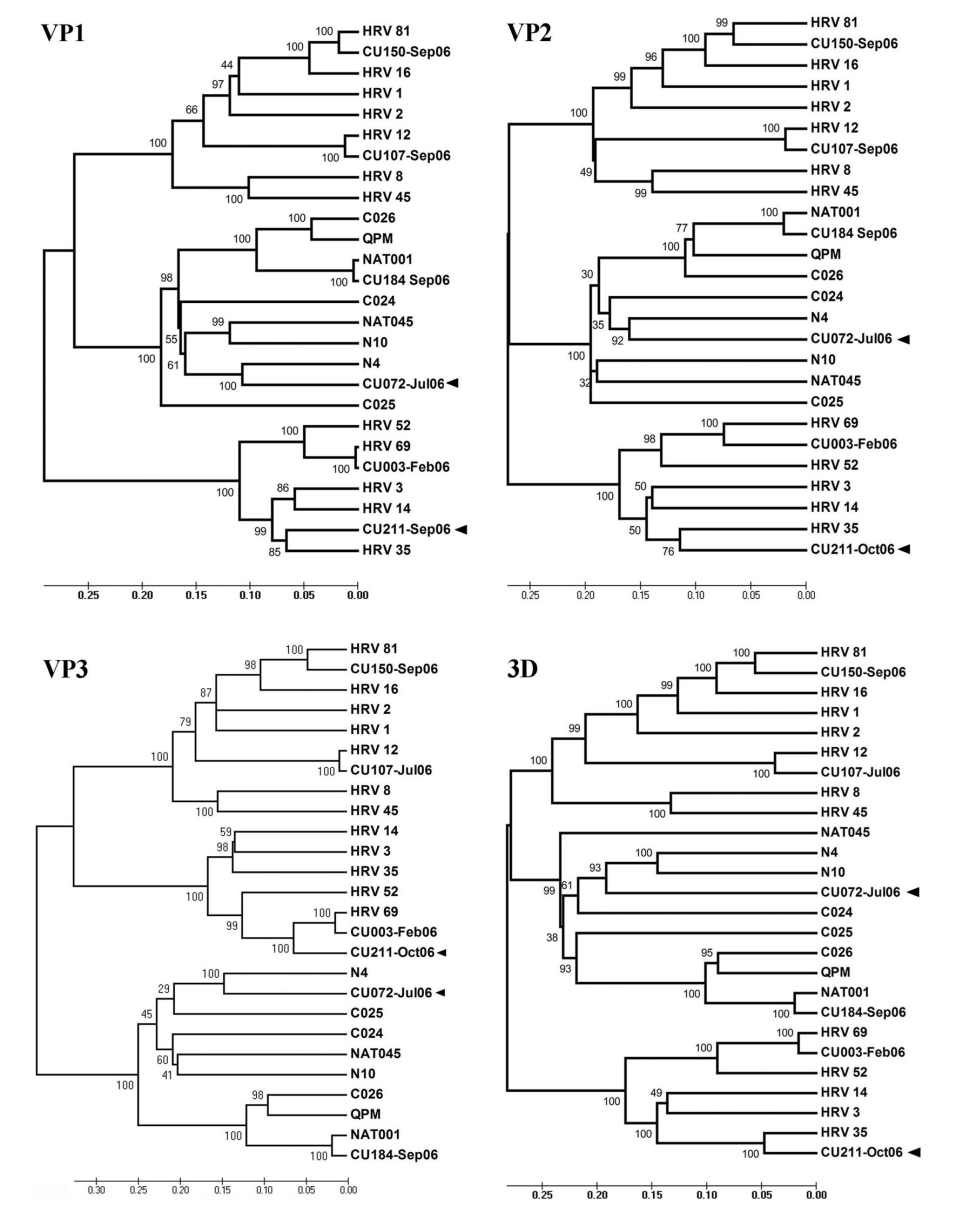
**Phylogenetic analysis based on deduced amino acid sequences of VP1-3 and 3D viral proteins of 6 identified strains compared with previously published prototypes**. Two new strains, HRV-CU072 and HRV-CU211, derived from our study are denoted by a black arrow. CU211 resulted from recombination between HRV-35 (major parent) and HRV-69 (minor parent). Tree constructions based on neighbor-joining method with 1,000 replicates.

In order to determine HRV diversity and evolutionary characteristics, potential recombination events in the polyprotein gene were evaluated by comparison with all available HRV reference sequences. The results derived from a recombination detection program combined with similarity plot, bootscanning method (Figure 3), and phylogenetic relationship (Figure 5) suggested that the strain HRV-CU211 had arisen subsequent to multiple recombination processes within the HRV-B lineages. Most of HRV-CU211's coding sequence was similar to HRV serotype 35 (major parent: FJ445187) with 84% of pair-wise nucleotide sequence identity, while part of the capsid coding VP2 and VP3 regions (positions 766-1590 nt) were genetically related to serotype 69 (minor parent: FJ445151).

### G+C content

**Figure 6:**
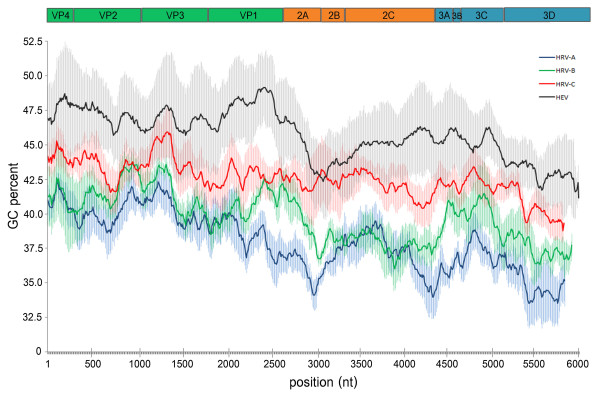
**Average G+C composition percentage along the ORF of HRVs and HEV. Each viral gene was depicted in relation to ORF arrangement**. Average values were computed from multiple sequence alignments of representative serotypes or strains with each species by using 500 bp sliding window and 10 bp increment size. Standard deviation (SD) value of each species' representative data was represented by the shaded area.

Compared with the closest relative, all HRV species exhibited a lower percentage of average G+C composition than other enterovirus members (Figure 6). HRV-A and HRV-B showed a relatively low average G+C content (38% and 39%, respectively) whereas HRV-Cs displayed the highest average value at 43%. HRV-C's 2A cysteine-type protease encoding region showed a unique G+C content more similar to enterovirus composition than other HRV species. In comparison the other enterovirus species, HEV-A and HEV-B, showed similar GC content (48%), polioviruses displayed 46%, HEV-C 45%, and HEV-D exhibited the lowest G+C content at 42%, closely related to HRV-C.

## Discussion

In this study, we have determined the complete coding sequences and summarized the molecular characteristics of a putative newly identified HRV-C strain. Furthermore, we have reported a new HRV-B member derived from intra-species recombination. In the absence of serological neutralization data of HRV-C, the HRV-C variants can be classified into 33 genetically-defined types based on divergence thresholds calculated from the distribution of pair-wise sequence distance. Results obtained from the HRV-CU072 strain showed it exhibited a low sequence similarity score (36% sequence divergence) and a distinct evolutionary phylogenetic relationship to the HRV-C criteria proposed by Simmonds et al. [23]. Several typical enterovirus and rhinovirus sequence characteristics are still conserved in HRV-CU072, such as potential utilization of the ICAM-1 protein as its specific receptor and possible resistance to synthetic pleconaril. However, this strain displayed some unique properties as for example, it uses a VP3/VP1 (N/D) cleavage site predicted by distinct alignment.

Several studies on rhinovirus, enterovirus and other picornavirus genera have examined variation across their genomes [39-41]. In HRV species, the structural proteins VP1, VP2 and VP3 and the nonstructural 3C and 3D proteins have been identified as diversifying selective regions that are thought to influence the evolution of HRVs. Although the capsid region is prone to high NS variability, the HRV-CU072 strain has conserved the essential motifs such as receptor interacting site and drug binding pocket along with other HRV-C members.

Our study compared nonsynonymous and synonymous substitution at the protein level of the HRV-CU072 strain with its phylogenetically closest relative (N4 strain) to elucidate the evolution of this newly identified strain. Analysis results suggested that the degree of sequence variation between them might not necessarily be ascribed to their genome size. Although the HRV-CU072 capsid region displayed high NS variation, the essential motifs such as receptor interacting site and drug binding pocket were conserved as in other HRV-C members. The VP4 capsid protein showed the highest sequence identity score compared with others. Due to its function as an internal surface protein VP4 is not involved in rhinovirus antigenicity. This might explain why the VP4 protein is highly conserved and shares familiar characteristics among the HRVs and HEVs. The analysis results revealed that the HRV-CU072 and N4 strains are descendants of a recent common ancestor via the purifying selection mechanism on the structural genes. In addition, this could suggest that the HRV-CU072 strain is not an N4 variant and might be a putative new HRV-C strain.

Based on our previous epidemiological study of semi-nested PCR covering the 5'UTR/VP2 region and VP4 phylogenetic classification [24], HRV-CU072 infection was detected in 3 of 289 NP suction specimens, accounting for 1% prevalence among the studied population without co-infection with other respiratory viruses. All of these patients had been diagnosed with acute lower RTI symptoms including pneumonia, acute bronchiolitis combined with wheezing and asthma exacerbation. Although the prevalence of the HRV-CU072 strain in the Thai population appears to be quite low, all patients presented with clinical symptoms associated with the development of a hyper-reactive airway disease. This may raise concern about the potential impact of this putative novel strain.

Ubiquitous recombination in enteroviruses and other picornavirus genera such as *Aphthovirus *and *Teschovirus *has been well established as an evolutionary driving force [42-46]. Despite its overall genetic similarity to HRVs, HEV recombination frequently takes place in either the nonstructural (mostly P2) region, or between the 5'UTR and adjacent capsid coding region. This results in a limited set of capsid genes responsible for HEV serotypes [44,46-48]. Many previous comparative studies have concluded that recombination in HRVs can occur throughout their genomes. The sites most favored for recombination have been frequently reported to occur in the noncoding and nonstructural regions [27,39,45,49,50].

In concurrence with the earlier reports, the results form PCM analysis described in this study also showed the overall recombination breakpoint of HRV species can randomly occur throughout the coding sequence. The PCM results of each HRV species illustrated that the different HRV species showed different degrees of phylogenic variation, representing a unique species-specific property. Interestingly, HRV species A exhibited a high degree of phylogenetic compatibility with each other within the capsid genes, 2C and nonstructural P3 regions. This indicates that the intra-species recombination processes of HRV-A were probably limited to these parts of the genome. In addition, all HRV-A members shared genomic characteristics conserved within the species and inter-species recombination was probably limited.

Huang et al., 2009 [36] and McIntype et al., 2010 [51] have reported that HRV-C showed evidence for inter-species recombination with HRV-A exhibiting 2 precise recombination hotspots in the 5'UTR and 2A gene. For the new species, HRV-C, PCM analysis results showed that sequence variations within HRV-C have been prone to accumulate in some genomic regions, particularly in the nonstructural 2A gene, as has been recently reported [49,51] and probably in the 3D coding gene which might influence the dynamic process resulting in intra-species C diversity. From our findings it could be concluded that the 3D gene encoding the RNA-dependent RNA polymerase is the site favored by HRV-C for recombination.

Only a few reports have indicated recombination in circulating strains. Recombination has recently been demonstrated between circulating heterogeneous HRV-A and some HRV-C strains. Palmenberg et al. [27] reported an intra-species recombination in HRV-A which resulted in the origin of a novel cladeD virus. Tapparel et al. [52] observed phylogenetic incompatibility in the 5'UTR, VP1 and 3CD regions of 2 HRV-A strains. Huang et al. [36] have also described HRV-A intra-species recombination events among 3 field strains with phylogenetic incongruency in the 5'UTR and VP4/VP2 regions and 2 HRV-C field strains have arisen from inter-species recombination with HRV-A. Our study suggests an infrequent recombination event among HRV-B lineages (HRV-CU211) identified from an acute lower RTI patient diagnosed with viral pneumonia with recombination breakpoints at the boundary of the capsid encoding VP2 and VP3 genes.

Although recombination events occurring in some parts of the different RNA genomes have not been recognized as a major mechanism for HRV evolution or as crucial for the large diversity of HRV circulating in humans, this process is still utilized for diversifying genome sequences. Furthermore, the detection of the recombinant strain in lower RTI patients may raise concern about the correlation between recombination and change in disease severity.

Studies on base composition in viral genomes can provide molecular information and thus contribute to understanding the efficient regulation of viral gene expression, codon usage bias, viral genome stability, and replication capability. Such information would also be relevant to elucidate their molecular evolution. Mutation pressure and composition constraint, particularly in G+C content, of the viral RNA genome are often considered important evolutionary genomic factors accounting for variations in codon usage among genes in different organisms [52-54]. In parallel with the molecular characteristics of HRV and HEV species, the average G+C content of their genomes has previously been described as a genomic factor to explain differences in RNA stability, optimal growth temperature, tissue tropism and also disease pattern.

In enteroviruses, a high G+C content of the viral genome is thought to be an essential factor for HEV's adaptive capability to replicate in various parts of the human body including respiratory tract, gastrointestinal tract, and central nervous system [52]. In contrast, the most closely related HRV species exhibited a lower G+C content than other enterovirus members which might reflect their adaptation to the lower temperature environment and sensitivity to the gastrointestinal tract's acidic pH. In this study, we found similar G+C content values of HRV-C and HEV-D coding sequences, contrary to the relatively low values in HRV-A and HRV-B species.

This may reflect HRV-C's capability to adapt to the higher temperature environment of the lower part of the human respiratory tract and thus differentiate it on some phenotypic level from other HRV species. This finding might also support several epidemiological studies on HRV in that HRV-C was more predominantly found in acute lower RTI cases than HRV-A and HRV-B and may significantly contribute to severe respiratory tract disease development, especially the exacerbation of asthma and wheezing. However, sequence analyses of other picornaviruses such as human hepatitis A viruses, hepatotropic members of the genus *Hepatovirus*, which replicate primarily in the gastrointestinal tract and spread to the liver causing liver failure and jaundice have shown a much lower G+C content [55]. To further understand this finding and investigate the mechanisms of virus-induced asthma exacerbations, HRV-C's mode of infection should be further investigated.

Little is known about the association between adaptive mechanisms and HRV evolution. Our results have provided information on the role of selection pressure and recombination mechanisms influencing the evolution of HRV. Further studies should be performed to better understand the clinical impact of each species on respiratory disease, epidemiology, their genomic characteristics, and the mechanisms controlling variation and evolution of this virus.

## Competing interests

The authors declare that they have no competing interests.

## Authors' contributions

PL carried out the molecular genetic studies, participated in the sequence alignment and drafted the manuscript. SP and KS participated in the sequence alignment. PL and YP participated in the design of the study and performed the data statistical analysis. YP conceived of the study in its design and coordination. All authors read and approved the final manuscript.
